# Solitary Metastasis of Colon Adenocarcinoma Mimicking Colloid Cyst of the Third Ventricle

**DOI:** 10.7759/cureus.15181

**Published:** 2021-05-22

**Authors:** Jaron M Hrushka, Joseph G Camarano, Thomas Frank, Gerald A Campbell, Aaron Mohanty

**Affiliations:** 1 Neurological Surgery, University of Texas Medical Branch, Galveston, USA; 2 Pathology, University of Texas Medical Branch, Galveston, USA

**Keywords:** choroid plexus tumor, colloid cyst, obstructive hydrocephalus, metastatic colonic adenocarcinoma, 3rd ventricular lesion, mr spectroscopy

## Abstract

Metastatic lesions to the choroid plexus, although far less common than colloid cysts, can present very similarly both symptomatically and radiographically. Choroid plexus metastases are most common in the lateral ventricles, however, when they occur in the third and fourth ventricles they may cause obstructive hydrocephalus typical of a colloid cyst lesion. Renal cell carcinoma is the most common primary cancer, but many rare primaries have been reported. When patients are presenting with symptoms typical of colloid cysts it is important to consider past oncological history and if past medical history is significant for cancer using MR spectroscopy may be valuable in distinguishing between cystic and metastatic lesions.

## Introduction

Metastatic lesions in the choroid plexus are exceedingly rare, accounting for less than 1% of clinically detectable cerebral metastasis, with 17.8% of reported cases occurring in the third ventricle. Renal cell carcinoma and lung adenocarcinoma are the most frequently reported primary carcinoma resulting in the intraventricular spread. Colloid cysts are far more common, accounting for roughly 2% of intracranial tumors and presenting with symptoms of hydrocephalus in roughly 50% of patients [1]. For this reason, an incidental obstructive third ventricular metastasis may easily be confused with a colloid cyst. We report a case of colonic adenocarcinoma metastasis to the choroid plexus of the third ventricle mimicking a colloid cyst and resulting in obstructive hydrocephalus.

## Case presentation

A 43-year-old female with a history of metastatic colon cancer presented to the emergency department with a three-week history of headaches and fatigue and a five-day history of nausea and vomiting. She denied any peripheral sensory changes, seizures, fevers, or chills. Seven years prior to the presentation, the patient was diagnosed with a moderately differentiated invasive adenocarcinoma obstructing the sigmoid colon for which she received a laparoscopic sigmoid resection. Since the initial presentation, she received 16 cycles of folinic acid, fluorouracil, and oxaliplatin (FOLFOX), four cycles of folinic acid, fluorouracil, and irinotecan (FOLFIRI), as well as stereotactic radiotherapy for recurrent metastases.

**Figure 1:**
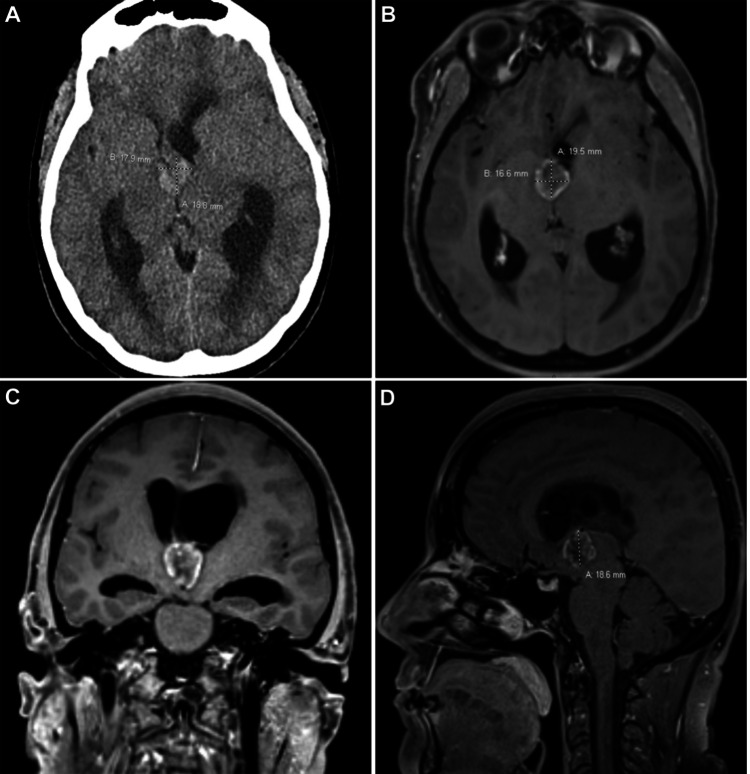
Preoperative Imaging Initial CT imaging concerning colloid cyst vs metastatic lesion with associated biventricular hydrocephalus (A: axial). Preoperative T1-weighted MRI with peripheral enhancement concerning neoplastic etiology (B: axial, C: coronal, and D: sagittal).

Non-contrast CT imaging showed a V-shaped hyperdensity measuring approximately 1.9 x 1.8 x 1.3 cm centered on the foramen of Monroe with moderate dilatation of the lateral ventricles (Figure 1A). Periventricular hypoattenuation was noted in the corpus callosum as well as diffuse sulcal effacement. CT findings along with her history of metastatic adenocarcinoma prompted a neurosurgery consultation. MRI confirmed an irregular peripherally enhancing mass, further raising suspicion for neoplastic etiology (Figures 1B-1D).

The patient underwent excisional biopsy of the mass with debulking via a left frontal burr hole. The mass was identified in the inferior aspect of the third ventricle, predominantly on the left side with extension through the foramen of Monro. Grossly, the lesion was yellowish-white, soft, and heavily vascularized. Diffuse bleeding from the tumor precluded endoscopic resection and a craniotomy was performed to achieve microscopic decompression and adequate hemostasis. The tumor was removed in piecemeal and an external ventricular drain (EVD) was placed in the lateral ventricle.

**Figure 2:**
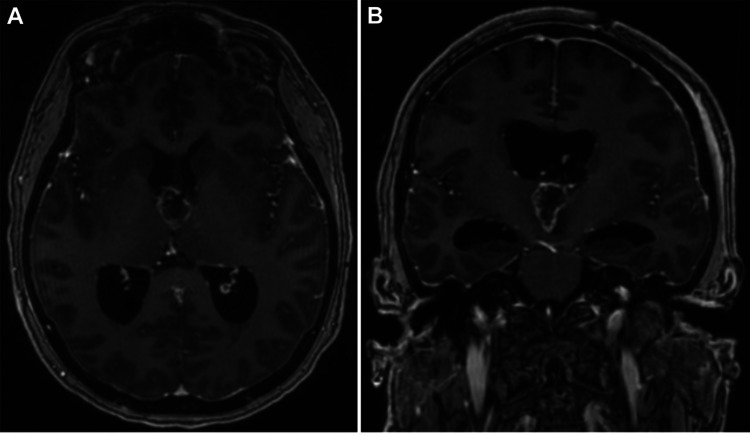
Postoperative Imaging Postoperative T1-weighted MRI showing peripheral enhancement throughout the resection cavity (A: axial and B: coronal).

Postoperatively, the patient exhibited improved mentation but with impaired short-term memory, which persisted following discharge. Follow-up MRI demonstrated thin peripheral enhancement throughout the resection cavity and CT imaging revealed progressive dilation of the lateral ventricles (Figures 2A, 2B). The patient suffered acute deterioration in mentation on attempts to wean from cerebrospinal fluid (CSF) drainage, and an endoscopic ventriculoperitoneal shunt placement with septostomy was performed. The patient convalesced well following shunt placement.

**Figure 3:**
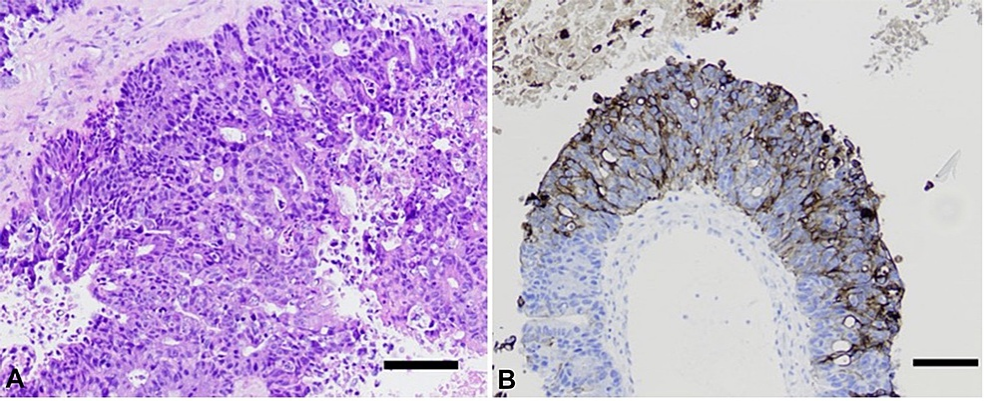
Immunohistology of Ventricular Lesion H&E stain with 100-micron scale bar (A), and cytokeratin 20 (CK20) stain consistent with metastasis from primary colon cancer with 100-micron scale bar (B). H&E: hematoxylin & eosin

Frozen section pathology of the resected mass was characteristic of adenocarcinoma with immunohistochemical positivity for cytokeratin 20 (CK20), consistent with metastasis from primary colon cancer (Figures 3A, 3B).

## Discussion

Metastatic ventricular system involvement is uncommon and, when present, is almost always associated with multiple brain metastasis. Metastases to the choroid plexus are exceedingly rare, with an estimated incidence of < 0.2% of cerebral metastases [2,3]. This low incidence is perplexing given the choroid plexus’ relatively high blood flow and lack of a blood-brain barrier, which are two major contributors to cerebral metastasis [4,5]. Although mechanisms are poorly understood, it is hypothesized that a rich collagenous stroma and tendency to accumulate amyloid render the choroid plexus inhospitable for metastasis [6]. It has also been suggested that specific tumor receptors which may otherwise aid in the metastatic spread may not be present in the choroid plexus, although this has not been sufficiently elucidated [7,8]. Additionally, reported cases demonstrate a disproportionate number of metastases originating from gastrointestinal and renal cell primary carcinomas, both of which typically demonstrate low malignancy and long latency [9].

Although isodense colloid cysts have been reported, diagnosis is typically suggested by location in the third ventricle and hyperdensity on CT imaging [10,11]. As the symptomatology of colloid cysts within or near the foramen of Monro depends highly on the extent of obstruction, large masses (i.e., ≥7mm) often require surgical excision, while smaller non-obstructive lesions may be monitored with serial CT scans [1]. Differentiating between a colloid cyst, ventricular metastasis, or primary central nervous system (CNS) neoplasm may be more difficult on a CT scan. As in the current case, intralesional enhancement on postcontrast MRI is more suggestive of a neoplastic etiology.

MR spectroscopy is also a viable option if the preoperative indication of neoplastic or non-neoplastic lesions is desired as it can reliably differentiate them. In the case of colloid cysts, the proteinaceous contents give a characteristic peak at 2 ppm identical to the neuronal marker N-acetylaspartate [12]. Primary gliomas and metastatic lesions on the other hand demonstrate increased peaks at 3.0 ppm and 1.3 ppm representing creatinine and lactate respectively, both indicators for increased metabolism. Additionally, intralesional necrosis is demonstrated with lipid peaks between 0.9 and 1.4 ppm [13]. Proper radiographic evaluation is also valuable in the determination of surgical options. While colloid cysts are typically avascular, metastatic choroid plexus lesions may be highly vascular [14]. For this reason, an endoscopic approach for resection of metastasis may be more difficult.

Much like obstructive colloid cysts, the presenting symptoms of choroid plexus metastasis are headache with nausea and vomiting if pathology is obstructive in nature [15]. As in our case, it is common for an isolated choroid plexus metastasis to present several years after the initial diagnosis of primary cancer [8]. A 2008 study reported an average discovery latency of over six years after the initial presentation in patients with ventricular metastasis [8]. In 10 of the 27 cases (37%), the discovery of an intraventricular mass led to the diagnosis of the primary tumor, further emphasizing the importance of a broad differential diagnosis in these patients.

The outcomes for choroid plexus metastases are typically poor. A study analyzing outcomes in 19 patients with renal cell carcinoma metastasizing to the choroid plexus showed a median overall survival of 2.8 years, with one- and five-year survival rates of 76.7% and 28.3%, respectively. Surgical resection resulted in lasting control of central nervous system symptoms without any associated long-term deficits [16].

## Conclusions

Despite the exceeding rarity of isolated choroid plexus metastasis, it should be included in the differential when a colloid cyst is suspected in a patient with a history of or risk factors for malignancy, especially of renal and colonic origin. In situations where the colloid cyst is obstructive in nature and surgical resection is indicated, the pathology report will reveal etiology. However, in asymptomatic cases where conservative management may be desired, further investigation may be warranted. In patients with prior oncological history, we recommend MR spectroscopy with follow-up biopsy if MR spectroscopy (MRS) is suggestive of neoplastic etiology.
